# Preinfection laboratory parameters may predict COVID‐19 severity in tumor patients

**DOI:** 10.1002/cam4.4023

**Published:** 2021-06-13

**Authors:** Alexander Kiani, Romina Roesch, Clemens M. Wendtner, Frank Kullmann, Thomas Kubin, Thomas Südhoff, Marinela Augustin, Markus Schaich, Clemens Müller‐Naendrup, Gerald Illerhaus, Frank Hartmann, Holger Hebart, Ruth Seggewiss‐Bernhardt, Martin Bentz, Ernst Späth‐Schwalbe, Peter Reimer, Ulrich Kaiser, Markus Kapp, Ullrich Graeven, Jens‐Marcus Chemnitz, Jörg Baesecke, Helmut Lambertz, Ralph Naumann

**Affiliations:** ^1^ Medizinische Klinik IV Klinikum Bayreuth GmbH Bayreuth Germany; ^2^ Comprehensive Cancer Center Erlangen‐EMN (CCC ER‐EMN) Erlangen Germany; ^3^ Technische Universität Dresden Dresden Germany; ^4^ Klinik für Hämatologie, Onkologie, Immunologie, Palliativmedizin, Infektiologie und Tropenmedizin München Klinik Schwabing Munchen Germany; ^5^ Medizinische Klinik I Klinikum Weiden Weiden Germany; ^6^ Klinik für Hämatologie & Onkologie Klinikum Traunstein Traunstein Germany; ^7^ Medizinische Klinik Klinikum Passau Passau Germany; ^8^ Klinik für Innere Medizin 5 Klinikum Nürnberg Nürnberg Germany; ^9^ Klinik für Hämatologie, Onkologie und Palliativmedizin Rems‐Murr‐Klinikum Winnenden Winnenden Germany; ^10^ Onkologische und Hämatologische Schwerpunktpraxis im Medizinischen Versorgungszentrum II Olpe Germany; ^11^ Klinik für Hämatologie, Onkologie und Palliativmedizin Klinikum Stuttgart Stuttgart Germany; ^12^ Klinik für Hämatologie und Onkologie Klinikum Lippe Lemgo Germany; ^13^ Zentrum für Innere Medizin Stauferklinikum Mutlangen Germany; ^14^ Medizinische Klinik V Sozialstiftung Bamberg Bamberg Germany; ^15^ Medizinische Klinik III Städtisches Klinikum Karlsruhe Karlsruhe Germany; ^16^ Klinik für Innere Medizin ‐ Hämatologie, Onkologie und Palliativmedizin Vivantes Klinikum Berlin Spandau Berlin Germany; ^17^ Klinik für Hämatologie, Internistische Onkologie & Stammzelltransplantation Evangelisches Krankenhaus Essen‐Werden Essen Germany; ^18^ Klinik für Hämatologie, Onkologie und Immunologie St. Bernward Krankenhaus GmbH Hildesheim Germany; ^19^ Klinik für Gastroenterologie, Hepatologie, Infektiologie, Hämatologie und Internistische Onkologie Sana Klinikum Hof Hof Germany; ^20^ Klinik für Hämatologie, Onkologie und Gastroenterologie Kliniken Maria Hilf GmbH Mönchengladbach Germany; ^21^ Klinik für Innere Medizin ‐ Hämatologie/Onkologie, Palliativmedizin Ev. Stift St. Martin Koblenz Germany; ^22^ Klinik für Hämatologie, Onkologie, Palliativmedizin St. Josefs‐Hospital Cloppenburg Cloppenburg Germany; ^23^ Fachabteilung Onkologie, Hämatologie & Palliativmedizin Klinikum Garmisch‐Patenkirchen Garmisch‐Partenkirchen Germany; ^24^ Klinik für Hämatologie, Medizinische Onkologie und Palliativmedizin Marien Kliniken Siegen Siegen Germany

**Keywords:** biomarkers, cancer, COVID‐19, neutrophils, SARS‐CoV‐2, tumor

## Abstract

**Background:**

Infection with SARS‐CoV‐2 leads to COVID‐19, the course of which is highly variable and depends on numerous patient‐specific risk factors. Patients with tumor diseases are considered to be more susceptible to severe COVID‐19; however, they also represent a heterogeneous group of individuals with variable risk. Identifying specific risk factors for a severe course of COVID‐19 in patients with cancer is of great importance.

**Methods:**

Patients diagnosed with solid tumors or hematological malignancies and PCR‐confirmed SARS‐CoV‐2 infection were included into the multicentric ADHOK (Arbeitsgemeinschaft der Hämatologen und Onkologen im Krankenhaus e.V.) coronavirus tumor registry. Detailed information about the patients’ cancer disease, treatment, and laboratory parameters prior to infection, was collected retrospectively. The outcome of the SARS‐CoV‐2 infection was graded according to the WHO.

**Results:**

A total of 195 patients (68% with solid neoplasms and 32% with hematological malignancies) were included in the registry. Overall, the course of the SARS‐CoV‐2 infection varied greatly, as 69% of all patients were either asymptomatic or encountered a mild to moderate course, while 23% of the cohort died from COVID‐19. In multivariable analysis, preinfection laboratory parameters (determined at least 10 days and a median of 21 days before the first documentation of SARS‐CoV‐2 infection) significantly correlated with severe course of the disease. Out of these, the absolute neutrophil count prior to infection showed the strongest association with COVID‐19‐related death.

**Conclusion:**

The course of COVID‐19 in patients with tumor diseases is highly variable. Preinfection laboratory parameters may aid to identify patients at risk for severe COVID‐19 at an early stage prior to infection with the virus.

**German Clinical Trials Register identification:** DRKS00023012.

## Graphical abstract

The course of SARS‐CoV‐2 infection in tumor patients is highly variable. Subgroups at risk for severe COVID‐19 are yet imprecisely defined. Laboratory parameters prior to infection, particularly pre‐infection neutrophils, may identify patients at risk for death.

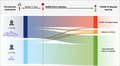

## 1 INTRODUCTION

Since its first detection in late 2019, the new coronavirus pathogen SARS‐CoV‐2 has spread from country to country causing a global pandemic, infecting more than 80 million people and accounting for more than 2 million deaths in 1 year.^1^ Infection with SARS‐CoV‐2 leads to the disease COVID‐19, the course of which is highly variable and depends on a number of patient‐specific risk factors, such as age, sex, diabetes, and other comorbidities.^2^, ^3^

Several studies suggest that tumor patients are at an increased risk of suffering a severe course of COVID‐19, with a mortality rate ranging from 10 to 30%.^3^, ^4^, ^5^, ^6^, ^7^, ^8^, ^9^, ^10^, ^11^, ^12^, ^13^, ^14^, ^15^ Increased age, male sex, the presence of comorbidities, poor performance status, and an active or progressive tumor disease are factors associated with an adverse outcome.^6^, ^7^, ^8^, ^9^, ^10^, ^11^, ^12^, ^13^, ^14^, ^15^ Furthermore, patients with lung cancer or hematological malignancies may be at higher risk than patients with other tumor entities.^6^, ^7^, ^8^, ^9^, ^10^, ^11^, ^16^

A challenging task for physicians involved in the care of cancer patients during the SARS‐CoV‐2 pandemic is finding the balance between the protection of vulnerable individuals and the necessity to continue antitumor therapy. Cancer patients are an extremely heterogeneous group of individuals with variable risk for a severe course of COVID‐19.^17^ Furthermore, they are on average older and harbor more comorbidities than patients without malignancies.^18^ Identifying factors that allow the estimation of the risks associated with the infection is therefore of utmost importance. Due to the high number of confounding factors, however, data from the above‐mentioned studies are partially conflicting and not yet conclusive.^17^

The ADHOK (Arbeitsgemeinschaft der Hämatologen und Onkologen im Krankenhaus e.V.) is a network of oncologists representing more than 100 non‐university hospitals in Germany. In an effort to obtain data about the impact of SARS‐CoV‐2 infection in German cancer patients, the ADHOK Coronavirus Tumor Registry was established. In particular, detailed information about the tumor disease and treatment, as well as laboratory parameters prior to infection, was collected in this registry. The aim was to identify patient‐specific risk factors for a severe course of COVID‐19. We report here the results of the first 195 patients.

## 2 METHODS

### 2.1 Study design and data collection

The ADHOK Coronavirus Tumor Registry is an ongoing observational and retrospective multicenter study, in which information about the course of a SARS‐CoV‐2 infection in tumor patients is collected. Patients with a PCR‐confirmed SARS‐CoV‐2 infection and a solid or hematological malignancy, either active or dating back up to 5 years prior to infection, were included. Tumor types and categories of the patients included in the study are specified in Table 1 and further detailed in Table S1.

**TABLE 1:** Patient characteristics and demographics

Characteristics	Patients n	%
**Total n**	195	(100)
**Sex**		
Male	113	(58)
Female	82	(42)
**Age [years]**		
Median (range, IQR)	73 (5–94, 18)	
<60	41	(21)
60–69	39	(20)
70–79	67	(34)
>80	48	(25)
**Any comorbidity**		
Yes	142	(73)
No	48	(25)
N/A	5	(3)
Arterial hypertension	103	(53)
Chronic cardiac disease	49	(25)
Diabetes mellitus	31	(16)
Chronic kidney disease	26	(13)
BMI >30	28	(14)
Chronic pulmonary disease	14	(7)
**Last ECOG prior to infection**		
0	79	(41)
1	46	(24)
2	25	(13)
3	11	(6)
4	4	(2)
N/A	30	(15)
**Tumor disease**		
**Solid**	**133**	(68)
Gastrointestinal tract	33	
Thorax	24	
Urogenital system	23	
Breast	19	
Pancreas or liver	12	
Head and neck	10	
Skin	7	
Sarcoma	3	
CNS	2	
**Hematological**	**62**	(32)
Lymphoma	38	
Multiple myeloma	11	
Acute leukemia	7	
MPN/MDS	6	
**Disease activity**		
Active / no remission	132	(68)
Partial remission	14	(7)
Complete remission	29	(15)
N/A	20	(10)
**Therapy (< 3 months prior to infection)**		
Patients with therapy	102	(52)
Patients without therapy	90	(46)
N/A	3	(2)
**Patients with local treatments**	**50**	(26)
Surgery	29	
Radiotherapy	29	
**Patients with systemic treatments**	**81**	(42)
Chemotherapy	47	
Targeted and/or antihormonal therapy	37	
Steroids (>10 mg/d > 5 days)	18	
Checkpoint inhibitors	10	
**Hospitalization**		
Hospital admission	158	(81)
Primary cause: SARS‐CoV−2 infection	110	
Primary cause: other reasons	48	
**COVID−19‐related deaths and mortality**		
Total cohort (n = 195)	45	(23)
In‐hospital cohort (n = 158)	43	(27)

Abbreviations: BMI, body mass index; CNS, central nervous system; IQR, interquartile range; MPN/MDS, myeloproliferative neoplasm/myelodysplastic syndrome; N/A, not available.

**FIGURE 1:**
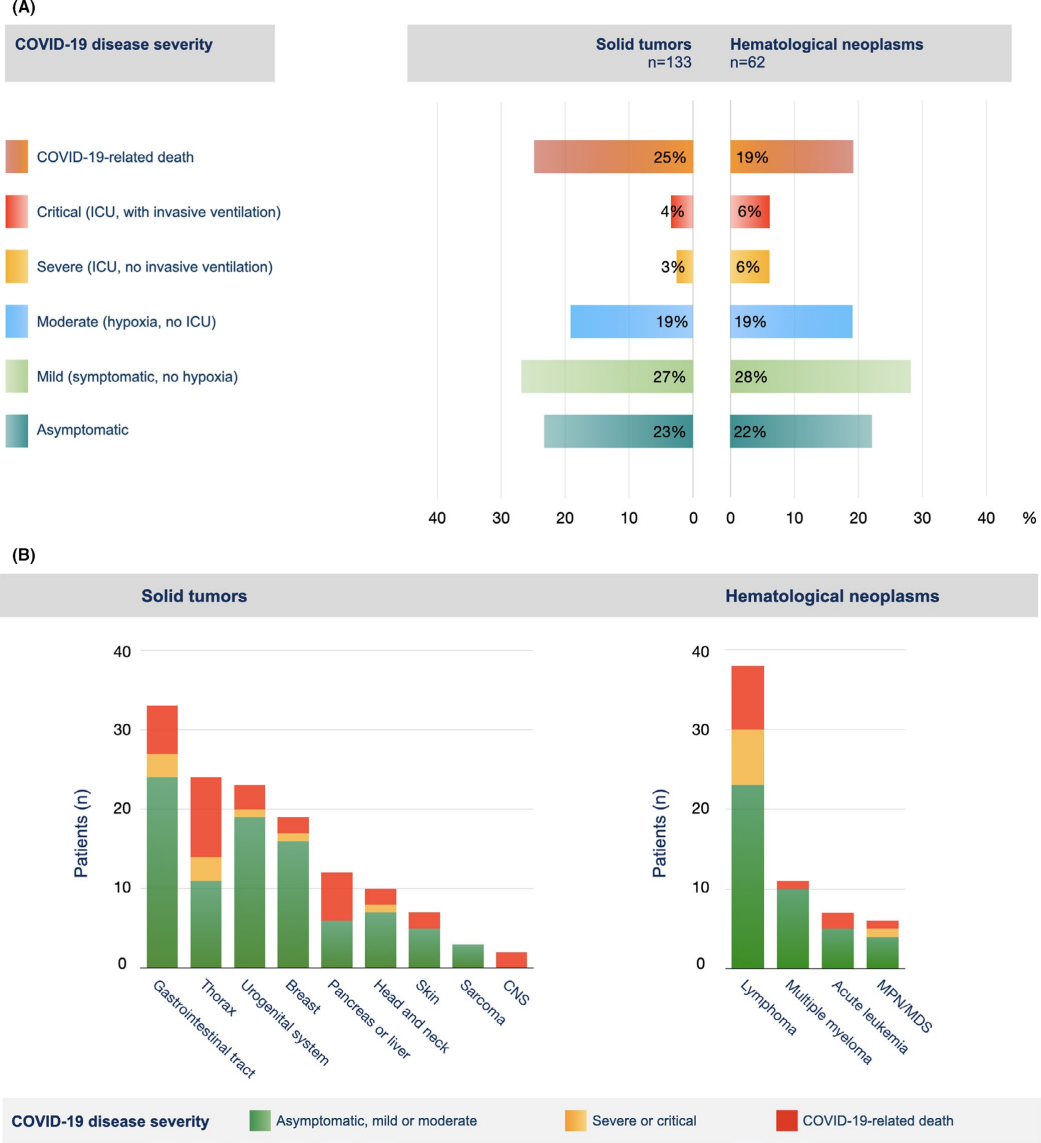
COVID‐19 disease severity in dependence of the underlying tumor disease. (A) COVID‐19 disease severity of patients with solid or hematological neoplasm was graded as either asymptomatic, mild, moderate, severe, or critical, according to the definitions of the WHO.^19^ COVID‐19‐related deaths are also indicated. (B) COVID‐19 disease severity is shown for patients with different tumor entities. Where indicated, patients with different tumor diseases were grouped according to the organ system involved. The heights of the bars correspond to the numbers of patients with the respective tumor entity. The colors within the bars represent the proportion of patients falling into the respective COVID‐19 disease severity category. ICU, intensive care unit; CNS, central nervous system; MPN, myeloproliferative neoplasm; MDS, myelodysplastic syndrome

Patients’ characteristics were documented anonymously into a specifically designed, web‐based form (https://core.adhok.de) by the treating physicians of the participating institutions or their delegates. The following categories were covered: demographic information, details of the tumor disease, details of the tumor treatment, pre‐ and post‐infection laboratory parameters, and outcome of the SARS‐CoV‐2 infection. Tumor disease in complete remission was designated as inactive. Preinfection laboratory values were collected at least 10 days preceding the first documentation of SARS‐CoV‐2 infection. COVID‐19 disease severity was categorized in accordance with the WHO definition into the following groups: asymptomatic, mild, moderate, severe, critical, and COVID‐19‐related death.^19^ Definitions of the categories are depicted in Figure 1A. Cause of death (COVID‐related or not) was assessed by the treating physicians.

The study was considered exempt from institutional review board (IRB) review by the ethics committee of the Bavarian State Board of Physicians (Bayerische Landesärztekammer) and has been registered in the German Clinical Trials Register (DRKS00023012).

### 2.2 Statistical analysis

Descriptive statistics of patients’ categorical variables are summarized as counts and percentages and continuous parameters as median (IQR and range). The Mann‐ Whitney *U* test or Kruskal–Wallis test was used to compare continuous data and the chi‐squared test or Fisher's exact test to compare categorical data. Logistic regression was used to estimate odds ratios (OR) and 95% confidence intervals (CI) for COVID‐19‐related death depending on clinical and laboratory prognostic factors in univariable and multivariable analyses. For univariable and multivariable analyses, continuous parameters were dichotomized by a median split. To include clinical and laboratory parameters in the multivariable analysis, the number of events available, strength of association in the univariable analysis, and the absence of collinearity between variables, assessed by chi‐squared test and Fisher's exact test for qualitative variables or Spearman's ρ for quantitative variables, were used. *p*‐values < 0.05 were considered significant. Statistical analyzes were performed using R version 4.0.3.

## 3 RESULTS

As of 31 December 2020, 195 patients with documented SARS‐CoV‐2 infection and solid tumor or hematological malignancy were included in the registry by 22 ADHOK institutions. A total of 158 patients were hospitalized; 37 patients were consulted in an outpatient setting. Only patients with a known outcome of SARS‐CoV‐2 infection (either discharge from hospital, death, or follow‐up of at least 30 days after infection) were included in the analysis. Patients’ characteristics are depicted in Table 1.

The outcome of the SARS‐CoV‐2 infection is shown in Table 1 and Figure 1. The mortality of COVID‐19 patients and fatality rate among hospitalized patients was at 23% and 27%, respectively. On the other hand, more than two thirds of the patients remained asymptomatic or had mild to moderate symptoms of the disease (Figure 1A). No notable difference was observed between patients with solid tumors and hematological neoplasms (Figure 1A). However, the course of the infection varied considerably between patients with different tumor entities (Figure 1B).

We then assessed potential risk factors for the outcome of SARS‐CoV‐2 infection in our registry. Age, ECOG performance status, and activity of the underlying tumor disease were significantly associated with COVID‐19‐related mortality in univariable analysis (Table 2). In contrast, antitumor treatment within 3 months or 3 weeks prior to infection did not have a significant effect on infection outcome.

**TABLE 2:** Univariable analysis of the correlation between clinical parameters and COVID‐19‐related death^a^

	Patients n / events n	Mortality rate	OR^b^	95% CI^b^	*p*‐value^c^
**Sex**	**195**				0.315
Female	82/16	20%	1		
Male	113/29	26%	1.42	0.71–2.84	
**Age**	**195**				**0.033**
<70 years	88/14	16%	1		
>70 years	107/31	29%	2.16	1.06–4.38	
**Last ECOG prior to infection**	**165**				**0.029**
0–2	150/32	21%	1		
3–4	15/7	47%	3.36	1.13–9.98	
**Disease activity**	**175**				**0.028**
Inactive	29/2	7%	1		
Active	146/41	28%	5.27	1.12–23.18	
**Solid vs. hematological neoplasms**	**195**				0.401
Solid	133/33	25%	1		
Hematological	62/12	19%	0.73	0.35–1.53	
**Any treatment (<3 months prior to infection)**	**192**				0.160
No	90/17	19%	1		
Yes	102/28	28%	1.62	0.83–3.20	
**Any treatment (<3 weeks prior to infection)**	**102**				0.210
No	13/3	23%	1		
Yes	89/25	28%	1.30	0.89–3.79	
**Systemic therapy (<3 months prior to infection)**	**102**				0.928
No	21/6	29%	1		
Yes	81/22	27%	0.93	0.56–1.95	
Chemotherapy (alone or in combination)	47/14	30%	1.06	0.91–3.20	0.094
Other systemic therapies (without chemotherapy)	34/8	24%	0.77	0.30–1.20	0.059
**Systemic therapy (<3 weeks prior to infection)**	**81**				0.493
No	26/8	31%	1		
Yes	55/14	26%	0.77	0.50–1.30	
Chemotherapy (alone or in combination)	34/10	29%	0.94	0.70–2.80	0.130
Other systemic therapies (without chemotherapy)	21/4	19%	0.57	0.50–1.10	0.280

Abbreviations: 95% CI, 95% confidence interval; OR, odds ratio.

^a^ The correlation between potential risk factors and the outcome of SARS‐CoV‐2 infection and COVID‐19‐related death was tested in univariable analysis.

^b^ Logistic regression was used to estimate odds ratios and 95% confidence intervals for COVID‐19‐related death depending on clinical prognostic factors.

^c^ Statistical significance was determined by logistic regression using the Wald test. *p*‐values < 0.05 (printed in bold) were considered statistically significant.

**FIGURE 2:**
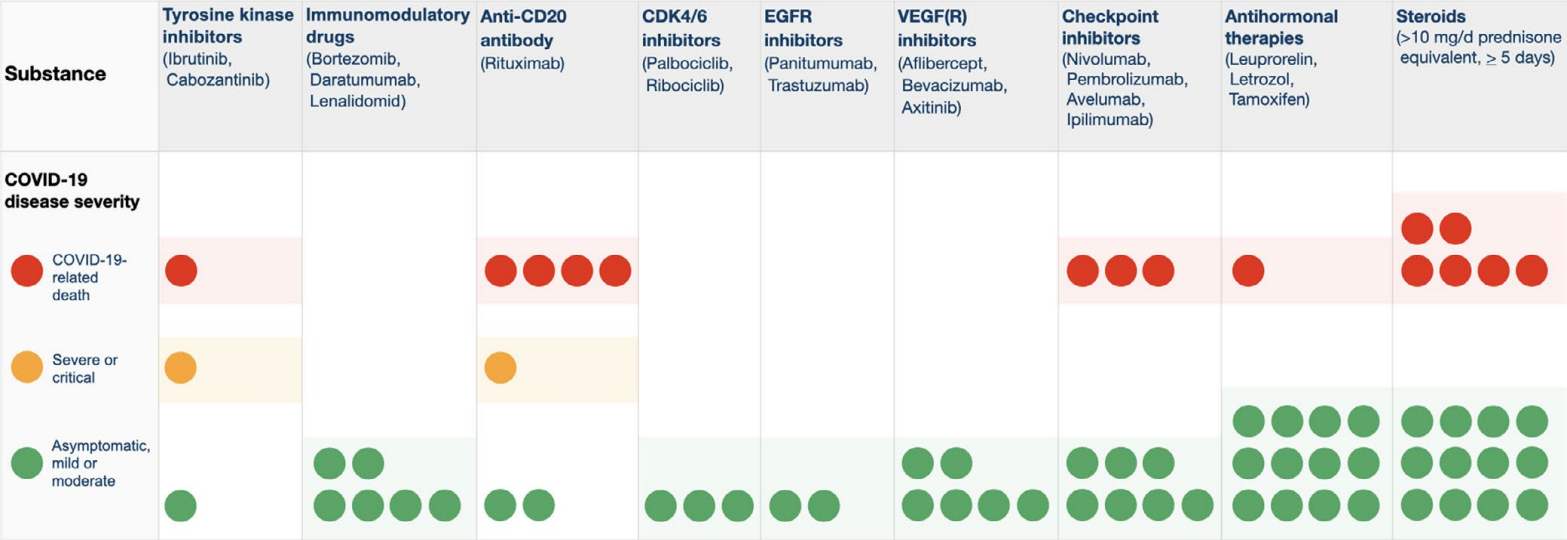
COVID‐19 disease severity in patients treated with various antitumor drugs. Patients treated with targeted, immunomodulatory or antihormonal drugs, as indicated, were grouped on the basis of the substance they received. COVID‐19 disease severity was graded as in Figure 1 and is shown for each subgroup. Each colored dot represents the treatment of a single patient. The three patients treated with cdk4/6 inhibitors also received antihormonal treatments and therefore are represented by dots in both categories

Systemic agents used for the treatment of cancer patients, such as kinase inhibitors, immunomodulatory drugs, monoclonal antibodies, or antihormonal treatments, may adversely affect the outcome of patients with SARS‐CoV‐2 infection. However, due to the large number of substances employed, subgroups of patients with identical treatments or treatment combinations are too small to detect statistical significance. To obtain an impression of the outcome of patients treated with different types of agents in our registry, we grouped them according to the treatment they received and assessed the course of COVID‐19 for each individual patient. As shown in Figure 2, treatment with targeted agents as indicated was rarely associated with fatal complications of the infection, with the notable exception of the anti‐CD20 antibody rituximab (4 out of 7 patients died) and the prolonged use of steroids (6 out of 18 patients died).

**FIGURE 3:**
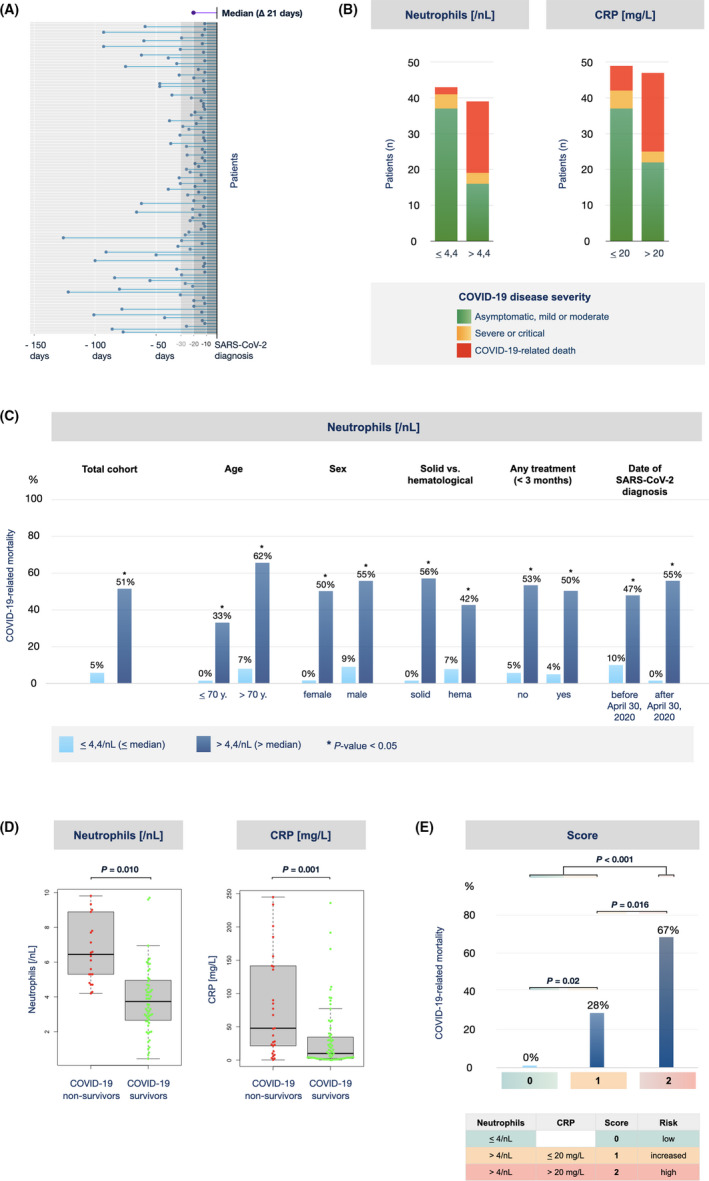
COVID‐19 disease severity in dependence of preinfection absolute neutrophil count and CRP. (A) Peripheral blood samples, that had been obtained at least 10 days (median 21 days) prior to the diagnosis of SARS‐CoV‐2 infection as part of clinical routine, were considered as preinfection samples and used for analysis. Each dot represents the time point of the blood sample of an individual patient with respect to his SARS‐CoV‐2 diagnosis. For each laboratory parameter, patients were divided into two groups using the median of all patients as a threshold. (B) COVID‐19 disease severity for patients with neutrophils (left) or CRP (right) below or above the median of all patients. The heights of the bars correspond to the numbers of patients of the respective group. The colors within the bars represent the proportion of patients falling into the respective COVID‐19 disease severity category. (C) COVID‐19‐related mortality for patients with neutrophils below or above the median of all patients. Shown is the mortality rate of the total cohort (left) or the subgroups indicated (right). *, *p* < 0.05. (D) Preinfection neutrophil counts and CRP values were compared in groups of patients surviving or not surviving COVID‐19. Shown are beeswarm box plots, with each dot representing one patient. *, *p* < 0.05. (E) Preinfection neutrophil counts and CRP values were used to calculate a score of 0, 1, or 2 points. Shown is the COVID‐19‐related mortality of patients grouped according to this score. *p*‐values < 0.05 were considered to be statistically significant. CRP, C‐reactive protein

Biomarkers of the peripheral blood, such as the neutrophil‐to‐lymphocyte ratio (NLR) or the C‐reactive protein (CRP), have been associated with COVID‐19 disease severity in patients both with or without cancer.^11^, ^20^, ^21^, ^22^, ^23^ However, as these parameters were usually obtained at the point of hospitalization for SARS‐CoV‐2 infection, they were likely influenced by the inflammatory reaction induced by the virus. We were interested if biomarkers available prior to infection were informative for the outcome of the disease and therefore analyzed laboratory values that were obtained at least 10 days (median 21 days) before the first positive SARS‐CoV‐2 PCR test was documented (Figure 3A). For each parameter tested, patients were divided into two groups using the median of all patients for that parameter as a cutoff. As shown in Table 3, several preinfection laboratory parameters were found to correlate with COVID‐19 mortality in univariable analysis. Interestingly, the preinfection absolute neutrophil count retained strong association with COVID‐19‐related death in multivariable analysis, with an odds ratio (OR) of approximately 14 (Table 4). A significant association, albeit to a lesser strength, was also apparent for CRP (OR 7.7) and lactate dehydrogenase (OR 2.8).

**TABLE 3:** Univariable analysis of the correlation between pre‐infection laboratory parameters and COVID‐19‐related death^a^

	Patients n / events n	Mortality rate	OR^b^	95% CI^b^	*p*‐value^c^
**Leukocytes prior to infection**	**120**				**0.002**
<6.8 /nL	59/6	10%	1		
>6.8 /nL	61/23	38%	5.35	1.99–14.39	
**Neutrophils prior to infection**	**82**				**<0.001**
<4.4/nL	43/2	5%	1		
>4.4 /nL	39/20	51%	21.58	4.57–62.45	
**Lymphocytes prior to infection**	**80**				**0.010**
<1.3 /nL	39/15	39%	1		
>1.3/nL	41/5	12%	0.22	0.07–0.69	
**Neutrophil to lymphocyte ratio (NLR) prior to infection**	**66**				**<0.001**
<4.0	34/2	6%	1		
>4.0	32/18	56%	20.57	4.19–100.89	
**Basophils prior to infection**	**56**				0.771
<0.04 /nL	28/9	32%	1		
>0.04 /nL	28/8	29%	0.84	0.27–2.64	
**Eosinophils prior to infection**	**57**				0.764
<0.1 /nL	30/10	33%	1		
>0.1 /nL	27/8	30%	0.84	0.27–2.59	
**Monocytes prior to infection**	**63**				0.750
<0.70 /nL	33/10	30%	1		
>0.70 /nL	30/8	27%	0.84	0.28–2.51	
**C‐reactive protein (CRP) prior to infection**	**96**				**< 0.001**
<20 mg/L	49/7	14%	1		
>20 mg/L	47/22	47%	5.28	1.97–14.13	
**Lactate dehydrogenase (LDH) prior to infection**	**72**				**0.005**
<235 U/L	37/6	16%	1		
>235 U/L	35/17	49%	4.88	1.63–14.62	
**Total calcium prior to infection**	**79**				0.335
<2.3 mmol/L	40/12	30%	1		
>2.3 mmol/L	39/8	21%	0.60	0.21–1.69	
**Urea prior to infection**	**71**				**0.023**
<38 mg/dL	36/8	22%	1		
>38 mg/dL	35/17	49%	3.31	1.18–9.24	
**Uric acid prior to infection**	**37**				0.909
<5.5 mg/dL	19/6	32%	1		
>5.5 mg/dL	18/6	33%	1.08	0.27–4.29	
**Creatinine prior to infection**	**109**				0.486
<1.0 mg/dL	55/14	26%	1		
>1.0 mg/dL	54/17	32%	1.35	0.58–3.10	

Abbreviations: 95% CI, 95% confidence interval; OR, odds ratio.

^a^ The correlation between pre‐infection laboratory parameters (obtained at least 10 days prior to SARS‐CoV‐2 infection) and COVID‐19‐related death was tested in univariable analysis. Laboratory parameters were dichotomized by a median split.

^b^ Logistic regression was used to estimate odds ratios and 95% confidence intervals for COVID‐19‐related death depending on pre‐infection laboratory parameters.

^c^ Statistical significance was determined by logistic regression using the Wald test. *p*‐values < 0.05 (printed in bold) were considered statistically significant.

**TABLE 4:** Multivariable analysis of the correlation between clinical and laboratory parameters and COVID‐19‐related death^a^

	OR^b^	95% CI^b^	*p*‐value^c^
**Sex**			0.201
Male	1		
Female	1.30	0.67–6.40	
**Age**			0.278
<70 years	1		
>70 years	1.50	0.76–3.78	
**Last ECOG prior to infection**			0.296
0–2	1		
3–4	2.61	0.76–8.90	
**Disease activity**			0.502
Inactive	1		
Active	4.25	0.48–37.61	
**Solid vs. hematological neoplasms**			0.594
Solid	1		
Hematological	0.51	0.13–1.78	
**Neutrophils prior to infection**			**0.010**
<4.4 /nL	1		
>4.4 /nL	14.00	1.06–31.90	
**Lymphocytes prior to infection**			0.399
<1.3 /nL	1		
>1.3 /nL	0.48	0.09–2.62	
**CRP prior to infection**			**0.019**
<20 mg/L	1		
>20 mg/L	7.73	2.63–38.81	
**LDH prior to infection**			**0.030**
<235 U/L	1		
>235 U/L	2.80	1.70–46.33	
**Urea prior to infection**			0.070
<38 mg/dL	1		
>38 mg/dL	2.00	0.98–5.24	

Abbreviations: 95% CI, 95% confidence interval; CRP, C‐reactive protein; LDH, lactate dehydrogenase; OR, odds ratio.

^a^ The correlation between clinical and pre‐infection laboratory parameters (obtained at least 10 days prior to SARS‐CoV‐2 infection) and COVID‐19‐related death was tested in multivariable analysis. Continuous parameters were dichotomized by a median split.

^b^ Logistic regression was used to estimate odds ratios and 95% confidence intervals for COVID‐19‐related death depending on clinical and laboratory prognostic factors.

^c^ Statistical significance was determined by logistic regression using the Wald test. *p*‐values < 0.05 (printed in bold) were considered statistically significant.

We then explored the relationship of preinfection neutrophils and CRP with infection outcome in our cohort in more detail (Figure 3B). Consistent with multivariable analysis, patients with neutrophils above the median of all patients showed a 10 ten‐fold higher mortality by COVID‐19 than those with values below the median (51% vs. 5%) (Figure 3C). The difference in survival was consistent throughout all subgroups tested, independent of whether the patients had an age below or above 70 years, were of female or male sex, suffered from a solid or hematological malignancy, were or were not treated for their tumor disease within the preceding 3 months, or were diagnosed with SARS‐CoV‐2 infection before or after April 30, 2020 (Figure 3C). Conversely, patients who died from COVID‐19 in our registry showed significantly higher levels of preinfection neutrophils and CRP than those who survived the infection (Figure 3D).

These results indicate that selected routine laboratory parameters determined at a time point prior to SARS‐CoV‐2 infection might aid physicians to identify tumor patients at risk for a severe course of COVID‐19. In order to develop an easy‐to‐apply score, we combined preinfection neutrophils and CRP values using the cutoffs indicated. As shown in Figure 3E, this score was able to separate three groups of patients with a significantly different outcome after SARS‐CoV‐2 infection in our registry.

## 4 DISCUSSION

Caring for cancer patients during the SARS‐CoV‐2 pandemic represents a challenge for oncologists and other health professionals. They must continuously balance the risks and benefits of exposing their patients to tumor‐specific treatments as well as balancing the weight of the decision behind pausing cancer therapy. The recognition of patients who are particularly vulnerable, and of treatments and circumstances which are particularly dangerous for these patients, is therefore of utmost importance.

We report here a series of 195 patients with cancer and documented SARS‐CoV‐2 infection and thereby summarize the experience of 22 German hospitals with the treatment of these patients. An important finding of our study is that SARS‐CoV‐2 infection in tumor patients does not necessarily lead to a severe or detrimental course of COVID‐19 as one might expect. Most cancer patients in our registry (more than two thirds) were asymptomatic or presented with only mild to moderate symptoms. Consistent with this notion, the in‐hospital mortality of 27% of patients in our registry is only slightly higher than the mortality rate of 22% observed in a cohort of more than 10,000 unselected patients treated in 920 German hospitals.^24^ However, as our registry is neither complete nor representative, all observed frequencies are influenced by patient selection. Nevertheless, our observation is in‐line with the recently published results of the European LEOSS register, in which the COVID‐19‐related mortality of matched patient populations with and without tumor diseases was found to be similar.^18^ Apparently, for most of the cancer patients the prognosis following SARS‐CoV‐2 infection is not dominated by the tumor diagnosis per se but determined by the same risk factors as for the general population, such as age, sex, and comorbidities.

On the other hand, there is a clear evidence that subgroups of tumor patients are particularly vulnerable to the infection, such as those with poor performance status or an active or progressive tumor disease.^13^, ^14^, ^16^, ^18^ Tumor patients differ largely with respect to their underlying tumor entity, their disease stage, their antitumor therapy, and a multitude of other potentially confounding factors. Due to this heterogeneity, even very large studies thus far were unable to provide consistent and robust evidence for additional risk factors potentially associated with an adverse outcome; for instance, with respect to specific antitumor drugs (or combinations thereof) or laboratory parameters.^17^, ^25^ Therefore, as much as possible data are needed to enable physicians to best possibly guide their patients through the crisis.

In our cohort, we confirm that the COVID‐19‐related mortality of patients with different tumor entities and systemic treatments is highly variable, even though numbers in subgroups are small (Figures 1 and 2). Surprisingly, the mortality of patients with lymphoma or myeloma, as for hematological malignancies as a group, was relatively low. Hematological malignancies have been associated with unfavorable prognoses in a number of studies,^6^, ^7^, ^8^, ^9^, ^11^, ^16^ but others report a better outcome.^15^, ^18^, ^26^ As outlined before, heterogeneity of patients and confounding factors are most likely responsible for this discrepancy and highlight the need to collect more data on subgroups that are particularly vulnerable.

Data on the influence of systemic therapies on mortality are particularly conflicting.^5^, ^6^, ^7^, ^10^, ^11^, ^12^, ^13^, ^14^, ^15^, ^17^ As in other studies,^11^, ^13^ in our cohort the impact of chemotherapy or other systemic therapies (as a group) on COVID‐19‐related mortality was not detected (Table 2). However, when the effect of single substances was examined in more detail, an indicator for adverse outcome was observed for lymphoma patients treated with the anti‐CD20 antibody rituximab and the prolonged use of steroids (Figure 2).

As a striking finding, the peripheral blood neutrophil count prior to infection turned out to be the strongest independent prognostic marker of death by COVID‐19 in our registry (Table 4). This observation is novel and needs to be confirmed in larger series. Of note, a severe or deadly course of SARS‐CoV‐2 infection has been associated with a dysregulation of inflammatory responses, cytokine storm, and activation of the coagulation cascade.^27^, ^28^, ^29^ Tumor and non‐tumor patients with severe COVID‐19 have been reported to have higher neutrophil counts, a higher neutrophil‐to‐lymphocyte ratio and higher levels of inflammation markers upon hospital admission than patients with mild disease.^11^, ^20^, ^21^, ^22^, ^23^ In contrast, preinfection peripheral blood samples were rarely available in the studies reported so far. In order to potentially identify biomarkers for a “predisposing state” of the host for severe COVID‐19 in our registry, we collected information on preinfection samples that based on an estimated incubation period of 4–7 days^30^—were obtained a median of 21 days before SARS‐CoV‐2 infection was documented. If confirmed, the combination of neutrophil count and serum CRP (determined at any given time point prior to SARS‐CoV‐2 infection), could be employed as an easy‐to‐obtain biomarker to identify tumor patients at particular risk for a severe course of COVID‐19. This would have potential implications not only for the protection of vulnerable individuals from SARS‐CoV‐2 infection, but also for the prioritization of vaccination strategies.

Interestingly, from a pathophysiological point of view, neutrophils and neutrophil extracellular traps (NETs) have been implicated in the pathophysiology of SARS‐CoV‐2‐mediated organ damage and mortality.^31^, ^32^, ^33^ Deterioration of patients with COVID‐19 has been observed after administrating neutrophil stimulation factors such as G‐CSF in a number of cases.^34^, ^35^ Of note, differences in neutrophil counts and CRP are well‐known prognostic factors for patients with tumor diseases.^36^, ^37^ We therefore propose a model in which tumor diseases to a variable extent (reflected by neutrophils and CRP) may induce a “pre‐inflammatory” state in the patients, that predisposes them to a hyperinflammatory reaction when they are infected with the virus. This detrimental reaction may be mediated, at least in part, by activated and potentially dysregulated neutrophils.^33^, ^38^

Our study is limited by its explorative and cross‐sectional nature, as well as the moderate number of patients included. Our findings at this point are therefore hypothesis‐generating and need to be confirmed in larger, independent, and (preferably) prospective trials. In any case, the results of our cohort will add to the collected experience of SARS‐CoV‐2 infection in tumor patients and hopefully aid physicians in the care for these patients during the pandemic.

## CONFLICT OF INTEREST

There is no conflict of interest to disclose for any of the authors.

## AUTHOR CONTRIBUTIONS

Alexander Kiani: conceptualization, data curation, formal analysis, funding acquisition, investigation, methodology, project administration, resources, supervision, validation, visualization, writing ‐ original draft. Romina Roesch: conceptualization, data curation, formal analysis, investigation, methodology, project administration, validation, visualization, writing ‐ original draft. Ralph Naumann: conceptualization, data curation, investigation, funding acquisition, writing ‐ review and editing. All other authors: data curation, investigation, resources, validation, writing ‐ review and editing.

## Supporting information

Table S1

## Data Availability

The data that support the findings of this study are available on request from the authors.
