# Supplementary material for: Preinfection laboratory parameters may predict COVID‐19 severity in tumor patients
Source: Cancer Med. 2021 Jun 13;10(13):4424–36. doi: 10.1002/cam4.4023 (PMC8267142; doi:10.1002/cam4.4023)
Supplement: Supplementary file 1 — Table S1 [file CAM4-10--s001.pdf]

Supporting information (Table S1): detailed listing of tumor entities included into the study.

Characteristics	Patients	n	%[a]
**Total n**		**195**	**(100)**
**Tumor disease**			
**Solid tumors (origin)**		**133**	**(68)**
**Gastrointestinal tract**		33	(25)
Rectum		13	
Colon		11	
Esophagus		5	
Stomach		2	
Anal		1	
Neuroendocrine		1	
**Thorax**		24	(18)
Lung		22	
Thymus		1	
Pleura		1	
**Urogenital system**		23	(17)
Prostate		11	
Kidney		5	
Bladder		5	
Germ cell (seminoma)		1	
Vulva		1	
**Breast**		19	(14)
**Pancreas or liver**		12	(9)
Pancreas		6	
Liver		6	
**Head and neck**		10	(8)
**Skin**		7	(5)
Melanoma		6	
Neuroendokrine (Merkel cell)		1	
**Soft tissue sarcoma**		3	(2)
**Glioblastoma**		2	(2)
**Hematological malignancies**		**62**	**(32)**
**Lymphoma**		38	(61)
Non-Hodgkin lymphoma		36	
Diffuse large B-cell lymphoma		11	
Chronic lymphocytic leukemia		9	
Mantle cell lymphoma		6	
Lymphoplasmocytic lymphoma		2	
T-cell lymphoma		2	
Follicular lymphoma		1	
NS		5	
Hodgkin lymphoma		2	
**Multiple myeloma**		11	(18)
**Acute myeloid leukemia**		7	(11)
**MPN/MDS**		6	(10)
Myelodysplastic syndrome		4	
Essential thrombocythemia		2	

Abbreviations: NS, not specified; MPN/MDS, myeloproliferative neoplasm/myelodysplastic syndrome

[a]Percentages in boldface refer to all patients, percentages in plain text refer to patients within the specific category of solid tumors and hematological malignancies, respectively.
